# Single Layer Broadband Anti-Reflective Coatings for Plastic Substrates Produced by Full Wafer and Roll-to-Roll Step-and-Flash Nano-Imprint Lithography

**DOI:** 10.3390/ma6093710

**Published:** 2013-08-27

**Authors:** Marieke Burghoorn, Dorrit Roosen-Melsen, Joris de Riet, Sami Sabik, Zeger Vroon, Iryna Yakimets, Pascal Buskens

**Affiliations:** 1 The Netherlands Organisation for Applied Scientific Research (TNO), De Rondom 1, 5612 AP Eindhoven, The Netherlands; E-Mails: marieke.burghoorn@tno.nl (M.B.); dorrit.roosen@tno.nl (D.R.-M.); zeger.vroon@tno.nl (Z.V.); 2 Holst Centre, High Tech Campus 31, 5656 AE Eindhoven, The Netherlands; E-Mails: joris.deriet@tno.nl (J.D.R.); sami.sabik@tno.nl (S.S.); iryna.yakimets@tno.nl (I.Y.); 3 Zuyd Hogeschool, Nieuw Eyckholt 300, 6419 DJ Heerlen, The Netherlands; 4 DWI an der RWTH Aachen e.V., Forckenbeckstrasse 50, 52074 Aachen, Germany

**Keywords:** anti-reflective, coating, nano-imprint lithography, roll-to-roll, durability, moth eye

## Abstract

Anti-reflective coatings (ARCs) are used to lower the reflection of light on the surface of a substrate. Here, we demonstrate that the two main drawbacks of moth eye-structured ARCs—*i.e*., the lack of suitable coating materials and a process for large area, high volume applications—can be largely eliminated, paving the way for cost-efficient and large-scale production of durable moth eye-structured ARCs on polymer substrates. We prepared moth eye coatings on polymethylmethacrylate (PMMA) and polycarbonate using wafer-by-wafer step-and-flash nano-imprint lithography (NIL). The reduction in reflection in the visible field achieved with these coatings was 3.5% and 4.0%, respectively. The adhesion of the coating to both substrates was good. The moth eye coating on PMMA demonstrated good performance in three prototypical accelerated ageing tests. The pencil hardness of the moth eye coatings on both substrates was <4B, which is less than required for most applications and needs further optimization. Additionally, we developed a roll-to-roll UV NIL pilot scale process and produced moth eye coatings on polyethylene terephthalate (PET) at line speeds up to two meters per minute. The resulting coatings showed a good replication of the moth eye structures and, consequently, a lowering in reflection of the coated PET of 3.0%.

## 1. Introduction

Anti-reflective coatings (ARCs) are used to lower the reflection of light on the surface of a substrate and enhance the transmission [1,2]. The areas of application for ARCs can be divided in two main categories: applications in which the ARC is used for aesthetic purposes and applications in which the ARC is used to improve the efficiency of devices. Examples of the first category are eyeglasses, museum and picture glass and displays [1,2,3,4]. Examples of the second category are lenses and devices that convert light to electricity and *vice versa*, like photovoltaic (PV) cells or light-emitting diodes (LEDs) in which the ARC contributes to a more efficient in- and out-coupling of light, respectively [1,2,5,6,7,8,9,10]. Much attention has been paid in recent literature to the use of ARCs in such solar and lighting devices [11]. Examples of improved in- and out-coupling of light through the use of ARCs on the device cover can be found for silicon, thin film and organic solar cells [5,6,7] and in lighting devices, like LEDs and their organic counterparts, OLEDs [8].

Three main coating concepts exist to lower the reflection and increase the transmission of a substrate. Multilayer stacks with alternating thin layers of a high and low refractive index can be used to create an interference filter [9]. They can be produced by vacuum/vapor deposition technologies, like sputtering or chemical vapor deposition (CVD), or by wet processing, e.g., using sol-gel chemistry [10]. The principle is based on destructive interference of the reflected light at the interfaces of the thin films in the multilayer stack. These coatings are typically robust and durable, but relatively expensive, due to the multiple coating and—in case of sol-gel coatings—curing steps; depending on the optical requirements of the ARC, the designs of two and more layers are reported to fine tune the optics [1,2,11,12,13,14]. These multi-layer interference filters are highly sensitive to process variations, like coating thickness and variations in refractive index. The optical performance can be tailored for specific needs, but is strongly dependent on the angle of incidence, since the optical path length in the layer stack increases with increasing angle of incidence. Multi-layer ARCs are applied to glass and polymeric substrates and have been on the market for several decades. Multi-layer ARCs are typically applied in high end markets in which the coating represents a high value. Examples of such markets are displays and picture and art glazing.

Secondly, an ARC can be realized using a coating of quarter wavelength thickness and a refractive index of $n=\sqrt{n_{s}\times n_{0}}$ , with *n_s_* as the refractive index of the substrate and *n*_0_ as the refractive index of the medium [1,2,15,16,17]. In the case of typical transparent polymeric substrates or glass with a refractive index of about 1.5 and air as medium, the ideal coating has a refractive index of 1.225. In order to lower the refractive index of a coating to that level, porosity has to be introduced in the coating material [1,2,15,16,17]. This type of quarter wavelength coating can be applied in one processing step and is, therefore, typically more cost-efficient than a multi-layer interference stack. The optical properties of such coating can be tuned; the reflection curve is typically V-shaped. Hence, a quarter wavelength ARC normally has a very intense reflection color and lacks broad-band anti-reflective properties. Additionally, the porosity in the system can make the coating mechanically less robust than multi-layer stacks. Furthermore, depending on the type, arrangement and accessibility of the porous structure, they can be susceptible to contamination. Porous quarter wavelength ARCs are commercially available. Quarter wavelength coatings are typically applied in markets in which the value of the coating is low to medium. Examples of such markets are solar cell covers and cover materials for LEDs and OLEDs.

The third coating concept is so-called nanostructured graded-index coatings [1,2]. The nanostructures in such coatings create a gradient in the refractive index normal to the substrate surface. This graded refractive index increases from the refractive index of the medium, in most cases, air, to that of the substrate. Moth eye nanostructures are the most common example for this technology [18,19,20,21,22,23]. The resulting coatings have broadband anti-reflective properties. Angular dependence of the reflection is for this type of coating very low, and the color neutrality is good to excellent. However, the decreased mechanical strength due to the nanostructured top coat can be a disadvantage.

Graded-index, moth eye ARCs can be made using, e.g., nano-imprint lithography (NIL). NIL was developed in the mid-nineties as a technology for producing micro- and nano-structured surfaces [24,25,26,27,28,29,30,31,32,33]. In this process, a nanostructured mold is used and either pressed into a softened polymeric surface (thermal NIL) or a wet UV-curable resist layer (step-and-flash NIL). In case of thermal NIL, the substrate is typically heated to about 50 °C above its glass transition temperature, the mold is pressed into the heated substrate surface and, after cooling, the mold is removed, leaving the polymer surface with a negative copy of the structure of the mold. In the case of step-and-flash NIL, a nanostructured mold is used and pressed into a wet, UV-curable resist. Then, the coating is cured through exposure to UV light, and after de-molding, the negative copy of the mold’s structure is left in the UV-cured coating layer. In the case of step-and-flash NIL, a positive copy of the mold can also be transferred to the UV-cured resist using a two-step procedure: transfer of the mold structure to a polymeric replica and transfer of the polymeric replica structure to the UV-curable resist. Both process variants are used to make micro- or nano-structured layers for use in a variety of devices and applications, e.g., devices for bio- and chemical sensing, transistors, random access memory patterns and integrated circuits [34,35,36,37,38,39,40,41,42]. In addition, NIL is often used to create nanostructured surfaces with an optical functionality [43,44,45,46,47,48,49], including moth eye ARCs [23,50,51,52].

Currently, however, moth eye ARCs are not commonly used in large-scale applications. The main reasons for this are the lack of suitable liquid coating materials, which are qualified for this application, and the lack of a simple, low-cost, high-volume, large-scale application process. Most commercially available coating materials—(imprint) resists—are designed for subtractive lithography processes in which the resist is used for structuring silicon wafers and does not end up in the final product [34,35,36,37,38,39,40,41,42]. Hence, typical resist materials are not designed for permanent application on substrates and, depending on the application, typically lack part of the required properties. In spite of a variety of studies performed in the past few years [53,54,55,56,57,58,59,60,61], NIL is not yet commonly used for large area, high-volume applications.

This article shows experimental results demonstrating the potential to eliminate both disadvantages to a large extent. This enables cost-efficient and large-scale production of durable nanostructured moth eye-type ARCs on polymer substrates.

## 2. Results and Discussion

### 2.1. ARCs Produced by Full Wafer Step-and-Flash NIL

To produce moth eye-type ARCs by full wafer step-and-flash NIL, we used the commercially available HT-AR-09 mold from Holotools. The mold was replicated in a hard polydimethylsiloxane (h-PDMS). Using the h-PDMS replicas, we made imprints in the commercially available UV-curable resist, Ormocomp, which has a refractive index of 1.535 at 500 nm and is available from Microresist Technology. This resist has been specifically designed for use in NIL. The imprints in the resist were made using the Nanoimprint Technology Platform from NIL Technology. The h-PDMS replica is pressed into the photosensitive coating material, which was applied using a roll bar, and cured with UV light through the replica. The approximate layer thickness of the Ormocomp coating is 15.4 µm. Finally, the h-PDMS replica is removed. The resulting coating layer has a moth eye structure identical to that of the HT-AR-09 mold. The residual layer thickness is about 15 µm. The residual resist layer can serve as a hard coat underneath the ARC. For regular single layer ARCs, the process involves two coating and curing steps: application of a transparent hard coat on the polymer substrate, curing of the hard coat, application of the ARC on the hard coat and curing of the ARC. Our process offers the potential to apply and cure the hard coat and top coat in one single step, which is a clear advantage over traditional single-layer ARCs on plastics.

#### 2.1.1. Replication and Imprint Process

**Figure 1:**
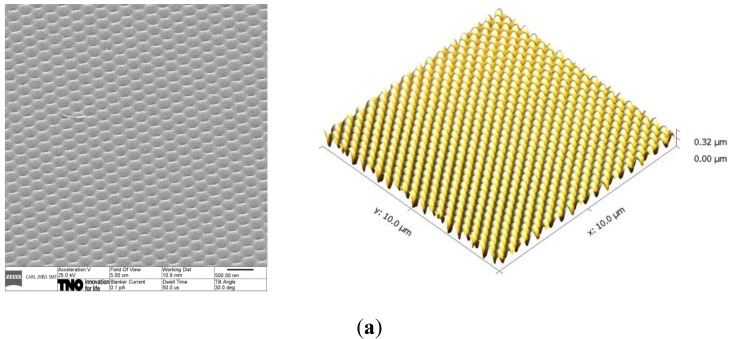

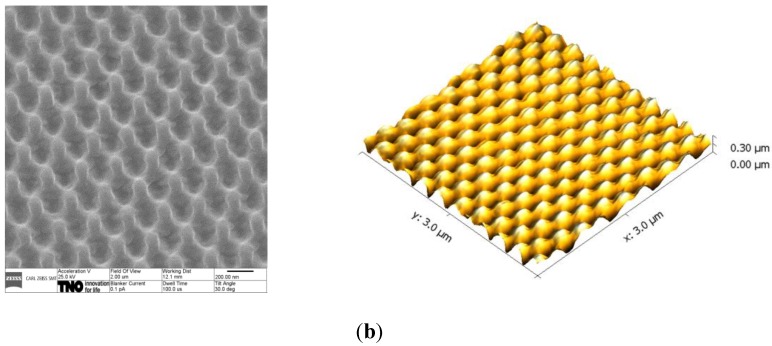
Helium Ion Microscopy (HIM) (left) and AFM (right) pictures of (**a**) h-PDMS replica; (**b**) Ormocomp on polymethylmethacrylate (PMMA).

The HT-AR-09 mold has the following specifications: the grating is a hexagonal array with a pitch of 250 nm, an average depth of 250 nm and a peak-to-peak distance of 300 nm. The surface structure of the h-PDMS replicas and the moth eye coatings on polymethylmethacrylate (PMMA, *n* = 1.49) made with these replicas are evaluated using Helium Ion Microscopy (HIM) and Atomic Force Microscopy (AFM). The results are displayed in Figure 1.

From the HIM and AFM images in Figure 1, we conclude that the surface nanostructure of the mold is well replicated in h-PDMS, and the surface nanostructure of the h-PDMS replica is well replicated in the Ormocomp coating. The pitch in lateral dimensions is 250 nm both for the h-PDMS and the moth eye-structured Ormocomp coating, which matches the original dimensions of the mold. The average structure depth for the structures on the h-PDMS replica and the Ormocomp coating is determined based on AFM data via analysis of a representative area of 1 µm^2^ and displayed in Table 1. As demonstrated by the average depth of the structures on the h-PDMS replica, the replication mold to replica is perfect. The average depth of the structures in Ormocomp is 284 nm. Although not perfect, the quality of the imprint from replica to coating is very good.

**Table 1:** Maximum depth and lateral pitch based on Atomic Force Microscopy (AFM) measurements. h-PDMS, hard polydimethylsiloxane.

Material	Lateral pitch (nm)	Average depth (nm)
Holotools mold	250	300
h-PDMS	250	300
Ormocomp	250	284

#### 2.1.2. Optical Properties of Moth Eye-Structured Ormocomp Coating

**Figure 2:**
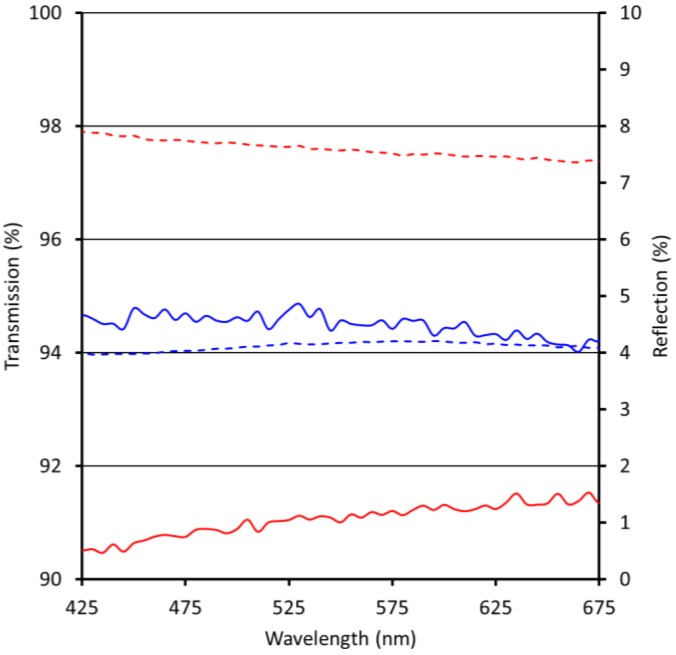
Reflection and transmission of uncoated polymethylmethacrylate (PMMA) (red) and PMMA with moth eye-structured Ormocomp anti-reflective coating (ARC) (blue); dashed lines = reflection, continuous lines = transmission.

**Figure 3:**
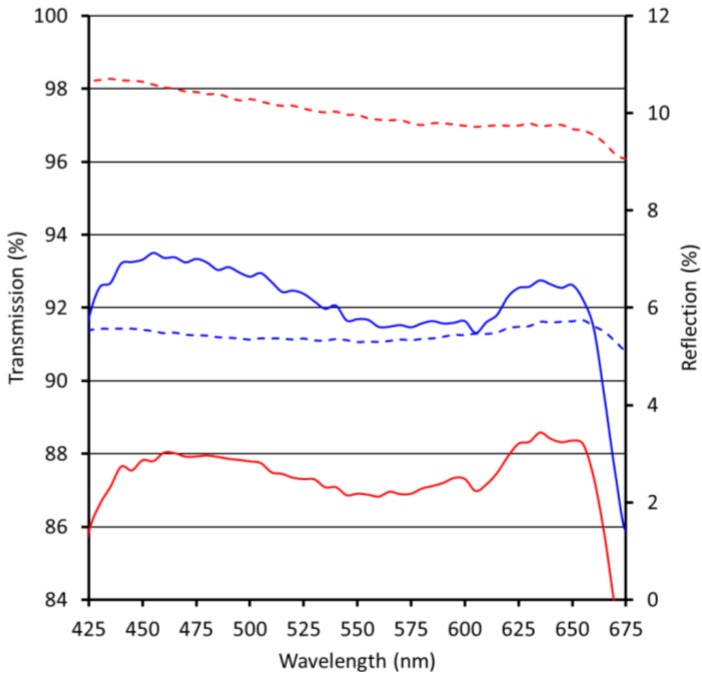
Reflection and transmission of uncoated polycarbonate (PC) (red) and PC with moth eye-structured Ormocomp ARC (blue); dashed lines = reflection, continuous lines = transmission.

Reflection and transmission measurements of single-side coated substrates are performed under near normal angle for the visible wavelength regime (VIS) between 425 and 675 nm. The result for the moth eye-structured Ormocomp coating on PMMA is displayed in Figure 2, on polycarbonate (PC, *n* = 1.58) in Figure 3.

The average reflection of PMMA in the visible spectrum is 7.6%. Using the moth eye-structured Ormocomp coating on one side of the substrate, the reflection is lowered by 3.5%. As displayed in Figure 2, the reflection of PMMA with a single-side imprinted coating layer is nearly constant in the visible spectrum, leading to broadband anti-reflective properties and a coating layer with a high degree of color neutrality. The average transmission of PMMA in the visible spectrum is increased by 3.4% after application of the ARC.

The average reflection for PC in the visible spectrum is 9.5%. The reflection is lowered by 4.0% after application of the moth eye-structured Ormocomp coating on one side of the substrate. As displayed in Figure 3, the reflection of PC with a single-side imprinted coating layer is nearly constant in the visible spectrum, leading to broadband anti-reflective properties with a high degree of color neutrality. The transmission is increased by 3.9%.

Both on PMMA and PC, the achieved anti-reflective effect matches the requirements for most commercial applications. The high degree of color neutrality of the coating matches the typical specification for application on displays.

#### 2.1.3. Adhesion and Scratch Resistance

To evaluate the adhesion of the Ormocomp coating to PMMA and PC, scotch tape tests were performed according to ISO2409:1992. For that purpose, pressure sensitive tape was applied to an area of the coating, which was cross-hatched with scratched lines. Adhesion is considered adequate if the coating is not pulled off by the tape when it is removed. For the moth eye-structured Ormocomp coating on both substrates, no coating material was removed during the scotch tape test, indicating good adhesion.

To evaluate the scratch resistance, pencil hardness tests according to ISO15184:1998 were performed for moth eye-structured and non-structured Ormocomp coatings on PC and PMMA. In all cases, three substrate samples were coated with Ormocomp resist, moth eye-structured using NIL and cured. Twenty-four hours after preparation, the pencil hardness of the coating was determined on three different positions per sample. Ergo, all values for pencil hardness are average values of nine measurements.

The pencil hardness is expressed as follows: the first value is the hardness of the hardest pencil that does not lead to a visible scratch on the surface of the coating; the second value is the hardness of the softest pencil that leads to a visible scratch on the surface of the coating. Approximate layer thickness of the Ormocomp coating without moth eye structures is 15.4 µm; the moth eye-structured Ormocomp coatings have a residual layer thickness of about 15 µm. The results are displayed in Table 2 and Table 3.

As displayed in Table 2 and Table 3, the pencil hardness of the non-structured Ormocomp coating layer on PC is HB-F, which is higher than the pencil hardness of the uncoated substrate (2B-B). The moth eye-structured Ormocomp layer, however, has a significantly lower pencil hardness (<4B). In case of PMMA as substrate, pencil hardness of the non-structured Ormocomp coating is similar to the pencil hardness of the uncoated substrate. For the structured Ormocomp layer, however, the pencil hardness is significantly lower (<4B). There are two explanations for the decrease in pencil hardness upon structuring of the Ormocomp coating: (1) the surface nanostructure makes the coating much more sensitive towards scratches and (2) surface scratches on an ARC are much more visible than on a coating or substrate with about 8% reflection in the visible spectrum. Upon scratching of the nanostructured surface, the anti-reflective properties are (partly) lost, leading to a reflective scratch on a non-reflective surface. For most commercial applications of ARCs, a pencil hardness of 3H or higher is required. With the current pencil hardness, the coating can only be applied for non-touch applications. Hence, further work is needed to improve the scratch resistance of the moth eye-structured coating. Lowering the friction on the coating surface through use of slip agents or stabilization of the surface structure by a thin coating layer conformally deposited over the moth eye-structured Ormocomp may improve the scratch resistance. Options for surface modification, however, are restricted by the optical requirements needed to achieve the anti-reflective effect.

**Table 2:** Pencil hardness test results of coatings on PC.

Coating	Imprint	Undamaged-damaged pencil hardness
Ormocomp	moth eye-structured	<4B
Ormocomp	non-structured	HB–F
Uncoated polycarbonate		2B–B

**Table 3:** Pencil hardness test results of coatings on PMMA.

Coating	Imprint	Undamaged-damaged pencil hardness
Ormocomp	moth eye-structured	<4B
Ormocomp	non-structured	2H–3H
Uncoated PMMA		3H–4H

#### 2.1.4. Durability of the ARCs

Accelerated ageing tests are performed on PMMA samples coated with non-structured and moth eye-structured Ormocomp to evaluate their durability. To get a first impression of the durability, three commonly applied accelerated ageing tests were performed: the damp heat test (1000 h at 85 °C and 85% relative humidity), the thermal cycling test (200 cycles between −40 °C and 85 °C) and the humidity freeze test (10 cycles between 85 °C and 85% relative humidity and −40 °C). The results of these durability tests are shown in Table 4. All samples were prepared and tested *in triplo*. The values in Table 4 are average values.

**Table 4:** Effect of durability tests on absolute differences in average reflection (%) in the visible—*i.e.*, 425 to 675 nm—regime. The change in reflection is presented in the table. (*∆R* = *R*_after_−*R*_before_)

Uncoated/coated PMMA	Δ*R* [%] Damp heat 1000 h 85 °C 85% RH	Δ*R* [%] Thermal cycling 200 cycles −40/85 °C	Δ*R* [%] Humidity freeze 10 cycles 85 °C 85% RH to −40 °C
Uncoated PMMA	+0.10	0.00	+0.07
Ormocomp non-structured	+1.11	+0.93	+0.57
Ormocomp moth eye-structured	+1.16	−0.08	−0.32

The adhesion of Ormocomp to PMMA is very good; no delamination is observed after the tests. As demonstrated by the results displayed in Table 4, the reflection of the moth eye-structured Ormocomp coating stays nearly constant in the humidity freeze and thermal cycling tests, but increases by 1.16% in the damp heat test. Overall, these test results match the specifications for most applications of ARCs. In most cases, however, further specific ageing studies are required.

### 2.2. Anti-Reflective Coatings Produced by Roll-to-Roll UV NIL

**Figure 4:**
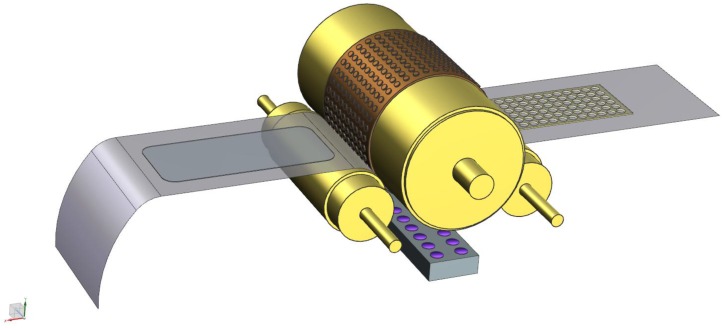
Roll-2-roll (R2R) imprint sub-system with the web direction from left to right: first, the non-structured, uncured resist on the web, the first roller, the drum containing the pattern with the UV LEDs below, the second roller and, finally, the structured, cured resist on the web.

**Figure 5:**
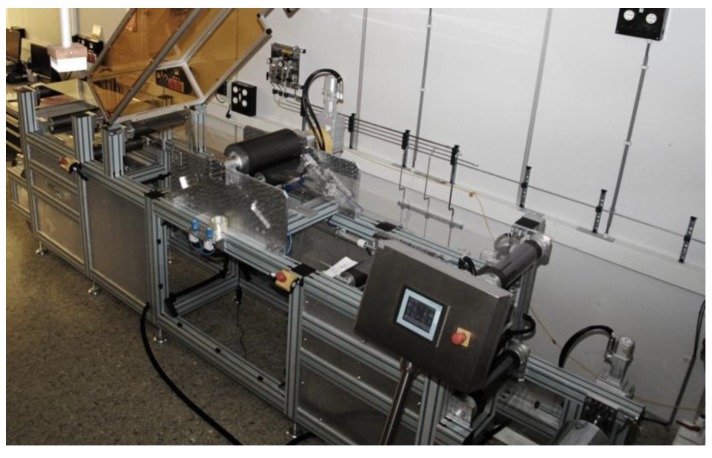
Picture of the R2R nano-imprint lithography (NIL) pilot line.

To apply the above mentioned ARC to large area, high-volume applications, it is necessary to use a continuous coating and structuring process. For that purpose, we designed process equipment to apply and structure coatings on foil in a roll-2-roll (R2R) fashion. For a schematic representation and pictures of the R2R pilot equipment, see Figure 4 and Figure 5, respectively. The system is fitted with a web tension management sub-system and an unwind-rewind sub-system. The business end is the imprint sub-system, which consists of a main drum and two contact rollers. The resist is deposited onto the web using a suitable R2R compatible technology, *in casu* droplet dispensing. The web and the resist are pressed onto the drum, which contains the moth eye-structured replica, by the first roller (imprint roller). In our specific case, the drum itself is not structured, but, instead, moth eye-structured PDMS replicas are fitted onto the drum. Hence, the resulting moth eye-structured ARC is not seamless. The resist is cured by high-powered UV LEDs, using the web tension to make sure the resist is cured in contact with the structure. After that, the release of the cured resist from the drum is facilitated by the second pressure roller, the delamination roller.

The tool has several adjustable parameters that are of influence to the imprint process, like line speed, web tension, UV intensity, imprint roller pressure (roller 1) and delamination roller pressure (roller 2). The tool is able to run at speeds in between zero, one and 3 m·min^−1^. The web width is 30 cm; the drum has a circumference of 60 cm. A UV blocking vented cover prevents any harmful gases from escaping to the environment (see Figure 5).

The tool is fitted with a droplet dispensing system. The thickness of the resist can be controlled by changing the pressure of the first roller, but is highly dependent on the viscosity of the resist. The tool is fitted with a high-power LED bar to cure the resist through the foil. The wavelength of the UV LEDs is 395 nm.

**Figure 6:**
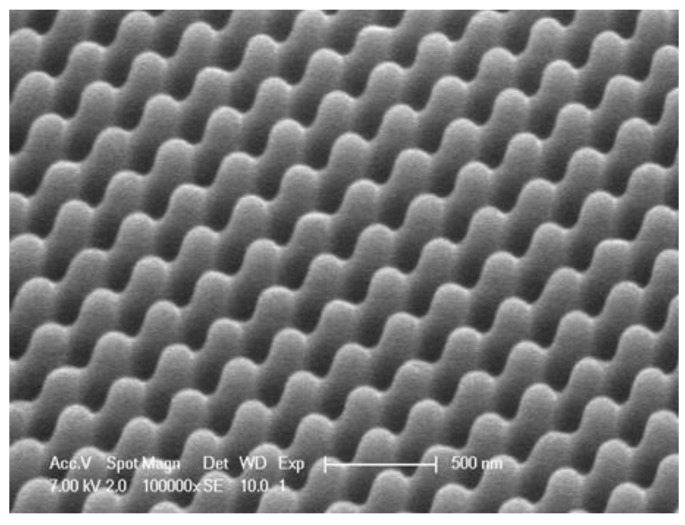
SEM image of moth eye-structured Ormocomp coating prepared using the R2R setup.

In the experimental set-up for producing moth eye-structured Ormocomp layers, we used identical h-PDMS stamps as used in the wafer-by-wafer experiments. The stamp was mounted on the imprint drum. The resist was applied to the web using the above-mentioned droplet dispensing system. After optimization of the process parameters, we were able to produce moth eye-structured Ormocomp coatings on polyethylene terephthalate (PET, *n* = 1.57). An SEM image of the moth eye-structured coating prepared at a line speed of two meters per minute using the above-mentioned R2R setup is displayed in Figure 6.

As displayed in this SEM image, the replication h-PDMS stamp to Ormocomp resist layer is very good. The average depth of the structures, as derived from cross-section SEM, is about 275 nm. The VIS reflection of the PET foil decreased from 7.05% to 4.0%; after application of the ARC, the transmission increased from 90.4% to 93.3%. The residual layer thickness of this sample was about 30 µm.

The cross-hatch adhesion test showed good adhesion of the coating to the substrate. The pencil hardness was <4B. Ergo, we were able to efficiently process Ormocomp on the R2R setup, displayed in Figure 4 and Figure 5. At a line speed of two meters per minute, we were able to properly replicate the structure of the h-PDMS stamp in the Ormocomp coating, leading to a decrease in the VIS reflection of 3.0%.

## 3. Experimental Section

### 3.1. Materials and Equipment

The substrate materials were 3 mm Lexan 9030 polycarbonate from Sabic and 2 mm Acrylite Solar IM20 polymethylmethacrylate from Evonik. PET foil was obtained from AGFA (125 µm thickness).

The HT-AR-09 mold was supplied by NIL Technology. This nickel mold contained moth eye nanostructures with a 250 nm pitch, 300 nm average height, a peak-to-peak distance of 300 nm and was supplied with an anti-sticking layer.

### 3.2. The Wafer-by-Wafer NIL Process

**Figure 7:**
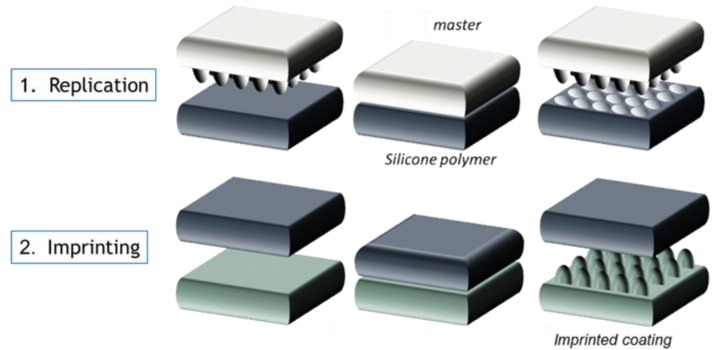
Schematic process overview of the replica preparation and structuring of the Ormocomp resist.

The moth eye nanostructures were applied through NIL. The process used consisted of two main steps: preparation of the replica and structuring of the Ormocomp resist, as illustrated in Figure 7.

#### 3.2.1. Preparation of the Replica

Nanostructure replication into a thin and flexible, UV-transparent silicone polymer was performed using the Holotools HT-AR-09 mold and a stack of two layers of polydimethylsiloxane (PDMS). A first layer of hard PDMS (h-PDMS) was applied on top of the mold. This material has a higher e-modulus compared to regular PDMS, but a much lower elongation at break. The material is quite brittle and requires a bilayer approach, using a second layer of softer PDMS [62].

The h-PDMS mixture was prepared by subsequently adding 30 mg of 1,3,5,7-Tetravinyl-1,3,5,7-tetramethylcyclotetrasiloxane, 6.8 g vinylmethylsiloxane-dimethylsiloxane trimethylsiloxy terminated copolymer, 2 g (25%–35% methylhydrosiloxane) of dimethylsiloxane copolymer and 30 mg of 1,3,5,7-Tetravinyl-1,3,5,7-tetramethylcyclotetrasiloxane. All four components were supplied by ABCR and used without purification. The materials were mixed using a speedmixer at 3500 rpm for 60 s. The h-PDMS was spin coated on top of the mold within 15 min after preparation, at 1000 rpm for 40 s. It was partly cured on a hotplate at 60 °C for 2 min.

Sylgard 184, supplied by Dow Corning, was used as the soft PDMS material as supplied. It was mixed in a mass ratio of part A:B 10:1 in a speedmixer at 2000 rpm for 60 s. The material was casted on top of the partly cured h-PDMS, until the mold was completely covered with a PDMS layer of approximately 0.5 mm. The stack was cured in a convection oven at 80 °C for 5 h. The replica was peeled off the mold and used in the NIL process.

#### 3.2.2. Structuring of the Ormocomp Resist

The UV-curable material used for imprinting was Ormocomp, supplied by Micro Resist Technology. It has a 100% solid content and was used as delivered by the supplier.

Prior to the wet film application, the substrates were dried in a convection oven for 10 min. The oven temperature for PMMA was 80 °C; for PC, 120 °C.

Ormocomp was applied with a 20 µm Bird applicator and prebaked in a convection oven at 80 °C for 15 min. The step-and-flash NIL process was performed using the Nanoimprint Technology Platform from NIL Technology. After the post-exposure bake in a convection oven at 80 °C for 15 min, the PDMS replica was removed from the sample.

The layer thickness was measured using a standard profilometer.

### 3.3. Characterization of Nanostructured Surfaces

#### 3.3.1. HIM

The images were made with the Zeiss Orion Plus HIM. This microscope is located on a special vibrational-free floor and placed in an in-house developed acoustic enclosure. The samples were scanned point by point with a sub-nanometer spot, with an acceleration voltage of 25 kV and a typical beam current between 0.1 and 1 pA. The images were recorded with an ET detector, collecting an amount of secondary electrons generated by beam sample interaction. A 5 nm carbon layer was deposited on the samples prior to analysis.

#### 3.3.2. AFM

A Nanosurf Flex AFM with high aspect ratio tips was used in tapping mode for AFM measurements.

### 3.4. Optical Characterization

A Shimadzu UV3600 spectrophotometer was used for total reflection and transmission measurements with an integrating sphere. The incident angle was 7°; the wavelength range was 300 to 1000 nm. The sampling interval was 5.0 nm and slit width, 20 nm.

### 3.5. Scratch Resistance Measurements

Pencil hardness tests were performed according to ISO15184:1998. In all cases, three substrate samples were coated with Ormocomp resist, moth eye-structured using NIL and cured. 24 h after preparation, the pencil hardness of the coating was determined on three different positions per sample. Ergo, all values for pencil hardness are average values of nine measurements. In the pencil hardness test, pencils of different hardness ranging from 9B (softest) to 9H (hardest) are moved over a coating surface using a well-defined carrier to guarantee constant weight. Under the applied weight, the soft pencil tips deform more than the harder ones, leading to a larger contact area, *A*. Since the force, *F*, is determined by the weight of the carrier and, therefore, constant, the contact pressure, *p*, changes gradually from low pressure (soft pencils) to high pressure (hard pencils).

### 3.6. Durability Tests

Three accelerated lifetime tests were performed on PMMA substrates with moth eye-structured Ormocomp, with the non-structured coating and with blank PMMA substrates. Reflection VIS was measured before and after testing. All samples were prepared and tested *in triplo*.

Three commonly applied accelerated ageing tests were performed: the damp heat test (1000 h at 85 °C and 85% relative humidity), the thermal cycling test (200 cycles between −40 °C and 85 °C) and the humidity freeze test (10 cycles between 85 °C and 85% relative humidity and −40 °C).

## 4. Conclusions

In conclusion, we prepared moth eye-structured Ormocomp coatings on PMMA and PC, using a wafer-by-wafer step-and-flash NIL process. The reduction in VIS reflection achieved with these coatings was 3.5% and 4.0%, respectively. The adhesion of the coating to both substrates was good. The moth eye-structured coating on PMMA demonstrated good performance in three prototypical accelerated ageing tests: the damp-heat, thermal cycling and humidity-freeze test. The pencil hardness of the moth eye-structured Ormocomp coatings on both substrates was <4B, which is less than required for most applications and needs further optimization. In addition, we developed a R2R UV NIL pilot scale process and produced moth eye-structured Ormocomp coatings on PET at line speeds up to two meters per minute. The resulting coatings showed a good replication of the moth eye structures and, consequently, a lowering in reflection of the coated PET of 3.0%. Further optimization of the process is currently ongoing in our laboratories. Ergo, we successfully demonstrated that the two main disadvantages of moth eye-structured graded index ARCs—*i.e*., the lack of suitable coating materials and a process for large area, high-volume applications—can be eliminated to a large extent, paving the way for cost-efficient and large-scale production of durable nanostructured moth eye-type ARCs on polymer substrates.
